# Manipulation of information type and task constraints during observational learning improve coordination but not accuracy and control parameters

**DOI:** 10.1371/journal.pone.0317270

**Published:** 2025-10-24

**Authors:** Davoud Fazeli, Fatemeh Jabbari, Leila Ghohestani, Hossein Taghizadeh

**Affiliations:** Department of Sport Sciences, Faculty of Education and Psychology, Shiraz University, Shiraz, Iran; Shahid Chamran University of Ahvaz, IRAN, ISLAMIC REPUBLIC OF

## Abstract

This study aimed to determine what information is used during observational learning and how this depends on task constraints using a mixed-design (between- and within-subjects) experimental approach. Specifically, the study aimed to examine whether full-body information or limited-body information enhances observational learning, and whether the influence of information is mediated by task constraints. For this purpose, participants (N = 48, mean age = 24 ± 5.3, male) were assigned to one of three demonstration conditions, each observing a point-light display (PLD) of a throwing action with varying kinematic information: full-body motion (BODY, 17 markers across major joints), right-arm motion (ARM, 4 markers on the throwing arm), or wrist-only motion (WRIST, 1 marker on the wrist). Each condition was divided into two sub-groups: one replicating the throwing action with a ball to a target (BODY-ball, ARM-ball, WRIST-ball) and one replicating the action without a ball, focusing on movement form (BODY-no ball, ARM-no ball, WRIST-no ball). These conditions manipulated the scope of visual kinematic cues and the presence of object-related task constraints to investigate their effects on motor learning outcomes. During the acquisition phase, participants performed 20 acquisition trials where a demonstration was shown five times on the first trial and then once again before each of the remaining trials. Twenty-four hours later, a retention test (5 trials with no demonstration) was performed. After retention, participants performed 10 further trials as re-acquisition. All participants observed a full-body PLD model in this period. Movement outcome, the similarity of intra-limb coordination, and wrist peak velocity in relation to the model were calculated. Results showed that in shoulder-elbow coordination, the BODY-ball group performed less like the model than the other groups in the retention test, all P < 0.05. In wrist-elbow coordination, a significant difference was observed in the ball condition, and the BODY group performed less like the model than the ARM and WRIST groups, all P < 0.05. Additionally, the no ball groups performed more like the model than the ball groups, all P < 0.05. In wrist peak velocity, the WRIST group performed less like the model than the BODY and ARM groups, all P < 0.05, and also, the ball groups performed more like the model than the no ball groups, all P < 0.05. These findings highlight that learning coordination patterns benefits from focused visual cues and minimal task demands, whereas learning velocity-related parameters depends on object interaction and more comprehensive kinematic information. This suggests that effective observational learning requires matching the type of visual information to the specific motor control demands of the task, offering insights for designing motor skill training protocols.

## 1. Introduction

Coaches use modeling and verbal instructions for conveying movement information to novice athletes, but demonstration is the most frequent way to convey this type of information. Observational learning was defined as a process whereby an observer attempts to replicate a behavior that has been demonstrated by another individual [1]. A three-phase neural model is proposed to explain how observational learning works: observation, acquisition, and response [2]. The first phase is defined as observing abstract stimuli and their association with specific bodily movements. The second phase involves the acquisition of rules, and the third phase relates to making the response. It is believed that all three phases include activation of brain areas similar those activated during physical performance [2]. The observation phase involves encoding abstract stimuli, activating the dorsal premotor cortex (PMd), right pars triangularis, right inferior parietal lobule, and posterior visual areas, mirroring physical performance’s PMd and parietal activation [3,4]. The phase of acquisition of motor rules activates the dorsal frontoparietal network (bilateral parietal lobes), fronto-striatal network (left dorsal striatum, dorsolateral/ventrolateral prefrontal cortices), and cerebellar networks, overlapping with physical practice’s sensorimotor and procedural learning regions [5,6]. The response phase executes actions, engaging the primary motor cortex (M1) and cerebellum, with error-monitoring in the middle cingulate cortex, posterior medial frontal cortex, anterior insula, and posterior superior temporal sulcus [7]. From a dynamic systems perspective, although systems are characterized by change, they are also limited by the extent of change that is possible. The human movement system is thought to be limited by three interacting constraints: the organism, the environment, and the task [8]. The organism refers to the characteristics of the individual. Environmental constraints arise in both the physical and the sociocultural environment of the performer. The task constraints include three types: the goal of the task, rules governing performance when imposed and instructional aids when provided (visual demonstration), and implements, tools, or equipment used during the performance of the task. Given the observed similarities between action observation and physical performance, it is possible that constraints, particularly task constraints, affect action observation [9].

Regarding the role of information, the visual perception perspective [10] is relevant. Based on the idea of direct perception [11], it is believed that a theory should focus on what information is used rather than how process of observation works [10]. Gibson [5] argued that the visual system has the ability to directly pickup information from the visual field. Based on this view, Scully & Newell [10] proposed that motion is essential to seeing. They believed that three perceivable types of motion are available during action observation. Absolute information describes the motion of a single element. Common information describes the motion common to all elements in the configuration relative to the perceiver. Relative motion refers to the motion of the all elements in the configuration relative to each other [12]. Scully & Newell [10] argued that during observation the observer picks up relative motion information (the motion of individual elements in the configuration relative to each other) and uses this for motion reproduction, particularly in the acquisition of coordination in early learning [10].

Several studies [13–18] have studied the role of relative motion information for observational learning by comparing the efficacy of video versus point light, dynamic displays (PLD). In a point light display, the structural and contextual information is removed, and only the points attached on joints are visible. It is argued that PLDs enhance the salience of relative motion information compared to videos, improving learning by eliminating all essentially distracting information [19]. Indeed, research showed that observing a ballet dance sequence in PLD format led to a closer approximation of a model’s coordination pattern than observation of the same information in a video [18]. This finding was replicated in studies addressing learning judo techniques [20] and knee rehabilitation [21]. Despite these positive findings, other attempts for showing the advantage of PLD rather than video display were not successful [13–17,22–24].

Researchers directly manipulated relative motion information in a kicking action to directly assess whether relative motion information is a critical parameter in learning from observation [25]. Participants saw either relative motion between joints of the kicking leg or just the motions of the foot (toe and ankle) or toe. If the relative motion is extracted and used for movement reproduction, then imitation should not be possible, or at least poorer in the impoverished conditions. The toe group was shown to approximate the model’s coordination as well as the relative motion groups, or better (i.e., hip- knee coordination). Moreover, after viewing a full-body model, they did not show any improvement in coordination. It was argued that observational learning is a more strategic rather than perceptually-driven process whereby the observer makes inferences about the action based on the trajectory of the end effector or task constraints when known (i.e., a ball and target). Researchers suggested that we should be cautious when ascribing significance to relative motion information in modeling processes, as its contribution may be overestimated. An observed convergence of an individual’s motion pattern toward a model post-exposure does not conclusively demonstrate that relative motion information within the display induced the coordinative shift. Through extensive movement experience, learners construct an abstract representation of their biomechanical structure, contextual dynamics, and inherent constraints, such as inter-joint dependencies. Observers can replicate actions using sparse informational cues, even in the absence of relative motion data [25].

These findings were confirmed in studies where participants copied a bowling action [26] and used video feedback while learning [27]. Endpoint trajectory information was useful for recognizing and reproducing new actions [26] and led to better dance performance than full-body information [27]. Latash [28] suggested that actions are planned based on their end trajectories, known as the working point. In line with this idea, other researchers found that the end-effector’s trajectory is key to differentiating actions [29]. If actions are planned by their working point, participants are likely to use this information during observation, and providing this information during observational learning should be enough to learn the motor skill. Despite these results, when a more complex cricket bowling action was shown in PLD format in terms of either the wrist of the bowling arm or a full-body display (both video and PLD) the full-body display resulted in better upper limb movement approximation [30]. However, participants reproduced the movement of the model’s bowling arm better than non- bowling arm, and there was not any difference between groups in non- bowling arm coordination profile. This finding supported the conclusion that the perceptual (visual) system has a priority for using information and the information from the end effector, which might be the whole limb or just the endpoint. This was confirmed through eye movement data in a subsequent study by this group [31]. In addition, these findings were also replicated in a study addressing learning a basketball jump shot [32].

Another factor affecting the observational learning is task constraints. There is some debate as to whether participants will perform more or less like the model when additional task constraints are present. Research evidence showed that in imitation of aiming movements, non-goal imitation (i.e., when the model moved to locations that were not delineated by targets) the movements were better approximated, than when target locations were shown [33]. Similarly, another study showed that both adults and children were more accurate in reproducing observed actions when task constraints were removed from the task (i.e., to bowl or not to bowl a ball) [34]. These results were replicated in index finger imitation [35] and when performers observed a joint pattern of basketball jump shot with either ball or no ball conditions [36]. However, other studies showed that movements were better approximated when the goal of the action was known (i.e., to kick a ball over a barrier rather than merely copy of lower leg action) [25]. One hypothesis is that task constraints are prioritized over informational constraints [9,37,38] such that task constraints might either hinder kinematic reproduction or aid reproduction if the movement to be imitated is complex (controlling multiple degrees of freedom simultaneously). According to the theory of goal-directed imitation [38], the imitator does not replicate the observed movement as a whole but instead breaks it down into distinct components. These components are organized hierarchically, with the most important aspect becoming the primary goal for the imitator. This suggests that people are more likely to imitate the final goal of an action rather than the method or style used to achieve it. It is believed that when learning a complex skill through observation, especially one focused on achieving an external goal, the movement pattern becomes secondary. The learner prioritizes reaching the external target and tends to neglect replicating the precise movement pattern, placing more emphasis on the outcome than the technique [38].

Another aspect that receives less attention is the timing of information delivery to the learner. It has been suggested that relative motion information is crucial in the initial stages of skill acquisition but becomes less significant as learning progresses [10]. If this holds true, observing a full-body demonstration during the later stages of acquisition should not lead to enhanced movement coordination for participants who were exposed to limited information during the initial phases [26]. In the current study, we wished to extend these findings to a task that had measurable outcome goals (i.e., physical constraints) that could be manipulated as well as manipulating the informational constraints. The aim was to determine how information from a model is prioritized when task constraints dictate a specific goal and how the information affects practice, as well as retention. These questions are important from both a theoretical and applied standpoint. The prioritizing of constraints affords insight into the interaction of the variables and any hierarchical ordering, as well as providing guidance as to how demonstrations are used to aid learning. As some of the previous studies showed, the information about movement does not matter much, given participants are attentive to the end effector [25]. However, task constraints seem to relate to what must be reproduced [38]. Therefore, information about the movement would relate to presence of task constraints. Previous studies did not consider different types of information (full body relative, restricted relative, and absolute) and task constraints in one experiment, hence performing a new experiment seems necessary. Moreover, while previous studies may have utilized tasks that were potentially familiar to participants, the present study introduces a novel task (back-hand throwing) to gauge how effectively observational learning facilitates the acquisition of a new coordination profile.

Understanding the relative importance of different types of information during observational learning can help coaches or practitioners to use observational learning process in a more useful way for motor learning and rehabilitation. Directing the learner’s attention to more useful information may results in higher learning during observational learning. To investigate how kinematic information and task constraints influence observational learning, we tested four hypotheses using a novel backhand throwing task with point-light display (PLD) demonstrations—full-body (BODY, 17 markers), right-arm (ARM, 4 markers), or wrist-only (WRIST, 1 marker)—and task constraints (ball: throwing to a target; no-ball: focusing on movement form).

**Hypothesis 1**: According to the visual perception perspective [10], it was hypothesized that observing relative motion information results in more similar coordination to the PLD model than absolute information. Accordingly, participants exposed to BODY information were expected to exhibit greater similarity to the PLD model than those observing ARM or WRIST information.

**Hypothesis 2**: According to the goal-directed imitation view [38], it was hypothesized that reaching an external target will be the main goal in ball-groups, accordingly, the coordination profile of no-ball groups should be more similar to the PLD model than ball groups. Consequently, coordination profiles in no-ball groups were expected to more closely resemble the PLD model than those in ball groups.

**Hypothesis 3**: According to the visual perception perspective [10], it was hypothesized that observational learning cannot enhance learning control related feature of the action (wrist peak velocity measure). If correct, no significant differences in wrist peak velocity should emerge between groups, nor should improvement be observed during acquisition.

**Hypothesis 4**: According to the visual perception perspective [10] it was hypothesized that observing relative information after the early acquisition phase cannot enhance coordination profile [10]. Thus, observing BODY information during the re-acquisition phase was not expected to enhance coordination profile or the control related parameters, such as wrist peak velocity.

## 2. Methods

### 2.1. Participants

Forty-eight male participants (mean age = 24 ± 5.3,) were randomly assigned to one of the six groups (n = 8/group). Previous studies used similar sample sizes [14,25,26]. The study started on 06/24/2018 and ended on 11/08/2019. Participants were eligible if they were right-handed, had normal or corrected-to-normal vision, and lacked formal experience in sports involving throwing actions. Individuals with neurological conditions affecting motor control, a history of upper or lower extremity injuries, or those not meeting any inclusion criterion were excluded. Participants were assigned to one of three demonstration conditions, each observing a point-light display (PLD) of an overhand throwing action with distinct kinematic information:

- BODY Condition: The PLD displayed 17 markers placed on major joints, presenting the full-body coordination of the throwing action. This condition provided comprehensive kinematic information, emphasizing the integration of proximal and distal movements.
- ARM Condition: The PLD showed 4 markers on the right arm (shoulder, elbow, wrist, and hand), isolating the kinematics of the throwing limb. This condition focused on arm-specific movement patterns, reducing extraneous whole-body information.
- WRIST Condition: The PLD included a single marker on the wrist of the throwing arm, highlighting the distal joint’s trajectory critical for fine motor control. This condition constrained visual information to the end-effector.

Each condition was divided into two sub-groups, resulting in six groups (as the videos presented to the participants were in PLD format, the ball was not directly observable. The experimenter manipulated the ball and no-ball conditions by verbally providing or withholding information about the action goal—throwing a ball):

- Ball Sub-Groups (BODY-ball, ARM-ball, WRIST-ball): Participants replicated the throwing action while holding a ball and aiming at a target, incorporating object interaction and task constraints.
- No-Ball Sub-Groups (BODY-no ball, ARM-no ball, WRIST-no ball): Participants replicated the throwing action without a ball, focusing solely on the movement form without object-related constraints.

For a visual illustration of the PLD demonstrations, please refer to the supplementary video materials. Also, a schematic presentation of the visual information available in each condition (BODY, ARM, and WRIST) is provided in Fig 2, section 2.3. It is important to note that the BODY groups (BODY-ball, BODY-no ball) watched similar information (information related to all 17 markers). The ARM groups (ARM-ball, ARM-no ball) also observed a similar demonstration (information related to the 4 markers placed on the throwing arm) and similar to two other demonstration conditions the WRIST groups (WRIST-ball, WRIST-no ball) observed a similar demonstration (information related to a single marker placed on the wrist of the throwing arm). However, the groups in each demonstration condition differed from having a ball or not having a ball.

**Fig 1:**
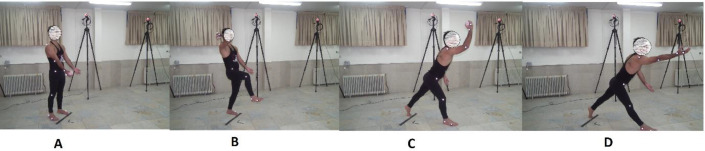
Backhand throwing action shown across four stills (A-D) depicting start position, backhand throw, release, and follow through, respectively.

The participants performed the experiment individually in a quiet laboratory. Participants provided written informed consent and were free to withdraw from the experiment at any time. All methods used in this study were approved by the institutional review board of Shiraz University.

### 2.2. Apparatus

Movement kinematics were collected using eight infrared motion analysis cameras (Osprey, Motion Analysis, Santa Rosa, U.S.A) sampling at 240 Hz. The demonstration was front projected onto a 1.8 × 1.3 m screen (Reflex, Australia) using a projector (INFOCUS, LP600, China) 6 m away from the start line. Participants in demonstration and ball groups were asked to throw an official softball toward a target positioned 5 m away in an area with 2 m × 2 m × 5 cm dimensions, filled with soft sand to record the landed ball position. In the center of the target was a red cross (the size of center of the cross was 10 cm).

### 2.3. Task and test- films

A back hand throwing action was shown that required movement of the right hand and right leg of the body simultaneously, as illustrated in Fig 1. The task consisted of four phases: starting position, backhand throw, release, and follow-through. In the starting position, participants stood in an anatomical stance (feet apart, hands at the sides of the body, palms facing outward) while holding the ball in their right hand. The backhand throw involved flexing the right elbow and right leg simultaneously. During the release phase, the right elbow was extended, and a step forward with the right leg was taken to throw the ball. The follow-through phase involved extending the hand and leg as much as possible.

**Fig 2:**
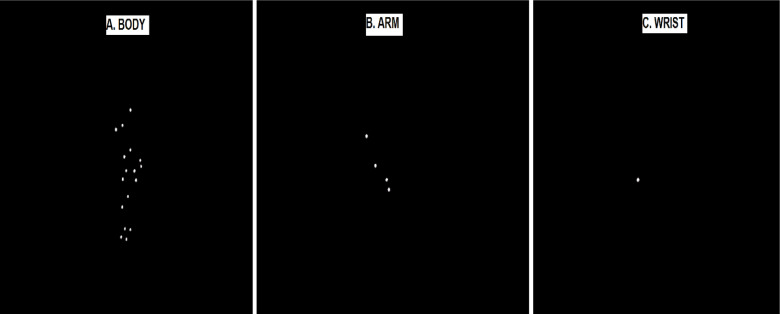
A screen shot from BODY (A), ARM (B), and WRIST (C) information.

A 24-year- old- male acted as the PLD model and was filmed from the sagittal plane. The filming took place after approximately 1000 trials across five days. The PLD model was positioned at the start line with his right hand at the front of his body, before throwing a ball to the target. On the last day of practice, a successful throw was chosen (i.e., one where the ball hit the target). This represented the kinematic profile adopted by the PLD model to achieve task success. Movement kinematics was collected from 17 reflective markers. The markers were placed on the major joint centers on the right and left side of the PLD model’s body, and these included the acromion process (shoulder), lateral epicondyle (elbow), ulnar styloid (wrist), the meta carpal head (finger), the greater trochanter (hip), the lateral condyle of the femur (knee), the lateral malleolus (ankle), the distal head of the fifth metatarsal (toe), and one on the center of the forehead [30,31]. A point light display of the PLD model’s throwing action was produced using the CORTEX software (Motion analysis, U.S.A). The point light display was edited to create three demonstration tapes (from the same action) where either all seventeen markers (BODY), four markers related to the right hand (ARM) or only one marker relating to the right wrist (WRIST) were presented (Fig 2). Participants viewed their respective demonstration from the sagittal plane.

### 2.4. Procedure

Before the experiment, seventeen reflective markers were placed on the major joint centers on both sides of each of the participant’s body. The positioning of these markers were the same as for the PLD model. In order to familiarize participants with the point-light stimuli, observers viewed a series of point light training videos showing a model executing a number of everyday actions, not related to the throwing action, in both point light and video format respectively [26]. Test sessions were conducted individually, in the laboratory (8 m × 8 m), and each test session lasted approximately 40 min. Before each trial, participants were asked to stand on the start line with their hands fully extended and in front of their body (like the PLD model’s start position, see Fig 1A).

### 2.5. Acquisition

**Fig 3:**
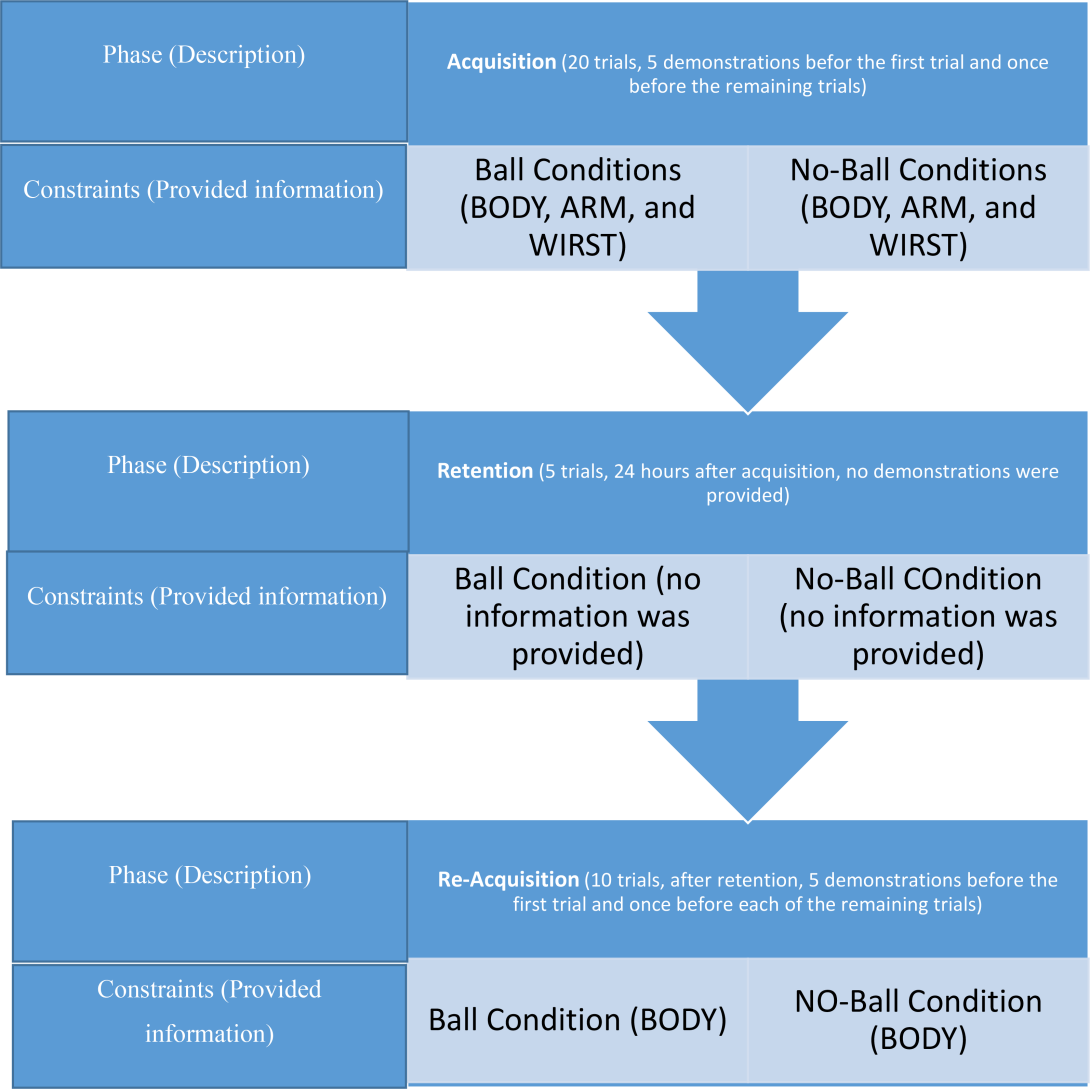
Schematic representation of experimental stages.

During acquisition, participants were asked to observe a point light display carefully and imitate the PLD model’s action as accurately as possible. The observers were not informed about the nature of the PLD model’s action (although this would be clear to participants in the “ball” groups). Participants performed 20 acquisition trials where a demonstration was shown five times on the first trial and then once again before each of the remaining trials. The BODY groups observed a full-body PLD model, the ARM observed a demonstration displaying four markers related to the right hand, and WRIST groups observed a wrist-only PLD model displaying only the wrist marker. The restricted information groups (ARM, and WRIS) were informed that the displaying markers are related to which part of the body. In addition, the BODY groups were informed that the markers’ positions on the model they observed were similar to the markers’ positions of their own bodies. All groups also were informed that they viewed the PLD model from side-on-view (sagittal plane). The ball groups were given a ball, and they were informed that the PLD model’s action resulted in the ball hitting the target. The no-ball groups were instructed to imitate the PLD model’s action, and nothing was told about the throwing action (Fig 3). According to the learning model provided by Newell [39], in the early phases of training, participants’ primary focus is on learning coordination. Therefore, during the acquisition phase of this experiment, participants aimed to replicate the coordination profile observed in the demonstration.

### 2.6. Retention

After the acquisition period- 24 h later- participants came to the laboratory and performed five trials as a retention test. Participants did not receive any demonstration in this period. In retention like the acquisition period, the ball groups received a ball and the same instruction to throw the ball into the target (Fig 3).

### 2.7. Reacquisition

After retention, participants performed 10 further trials. All of the groups observed a full-body point light model in this period. The demonstration order was like the acquisition period (i.e., five times before the first trial and once before each of the remaining trials). Like earlier periods (acquisition and retention), the ball groups received a ball and instructed to throw the ball into the target (Fig 3).

### 2.8. Dependent measure and data analysis

#### 2.8.1. Kinematics.

The start and end points of the movement were standardized. The start point was determined as the initiation of right elbow flexion (reducing the elbow angle in 10 consecutive frames), and the end point was determined as the maximum extension of the right elbow (the maximum elbow angle). The data were smoothed with a recursive 4th order Butterworth filter with a cut-off frequency of 7 Hz. A linear interpolation was performed to normalize this period to 100 data points [40] enabling comparisons across trials and with the PLD model.

To provide an index of similarity of intra- limb coordination of participant to the PLD model, a modified version of normalized root mean squared error (NORMS) was used [41]. In this approach, the PLD model’s trace was used as a criterion, and the disparity of each trial was calculated from PLD model’s criterion trace. Then, this score was normalized for a number of trials and excursion [42]. This approach called Normalized Root Mean Squared Difference (NORMS-D) [23]. A smaller NORMS-D score shows a closer approximation to the PLD model’s movement trace. The important aspect of this throwing action occurred on the right side of the body (right arm and leg). Because of this, we used of right shoulder-elbow and right wrist-elbow coordination to compare the intra- limb coordination between participants and PLD model. In addition, the wrist peak velocity was measured as a control-related feature, and again we calculated an absolute difference score in reference to the PLD model. A lower score would indicate a closer approximation to the PLD model [23,34].

In the acquisition, the kinematic data were computed from first three (ACQ1, t1-3), middle three (ACQ2, t 9–11), and last three (ACQ3, t 18–20) trials of acquisition. The resulting values were analyzed in a 3 model (BODY; ARM; WRIST) × 2 task condition (ball; no ball) × 3 block (ACQ 1, 2, 3) mixed design ANOVA with repeated measures on the last factor. In retention, data from the first three trials were analyzed in a 3 model (BODY; ARM; WRIST) × 2 Task condition (ball; no ball) factorial ANOVA. In reacquisition, the data computed from first three (RE- ACQ 1, t1-3), and last three (RE- ACQ 2, t 8–10) trials were compared with the last three trials of acquisition (i.e., ACQ3). These resulting data were analyzed in a 3 model (BODY; ARM; WRIST) × 2 task condition (ball; no ball) × 3 block (ACQ 3; RE- ACQ 1, 2) mixed design ANOVA with repeated measures on the last factor.

#### 2.8.2. Outcome error.

The X and Y coordinates of the ball’s landing position from the center were measured as centimeters (cm). By using these coordinates, the radial error was calculated, and then the mean was calculated for each block (see equation 1).


$$r=\sqrt{x^{2}+y^{2}}$$ (1)

Where r is the radial error, x is X coordinate of the landed ball, and y is the Y coordinate of the landed ball. The calculated radial error was converted to the meter scale.

In the acquisition, the data were analyzed using a 3 model (BODY; ARM; WRIST) × 3 block (ACQ 1, 2, 3) mixed design ANOVA with repeated measures on the last factor. In the retention, the data were compared in a one- way ANOVA for BODY, ARM, WRIST groups. Finally, in the reacquisition, the data were analyzed in a 3 model (BODY; ARM; WRIST) × 3 block (ACQ 3; RE-ACQ 1, 2) mixed design ANOVA. The P value = 0.05 was considered statistically significant. The partial eta squared was reported as effect size. Cohen (1988) has provided benchmarks to define small (η^2^_p_ = 0.01), medium (η^2^_p_ = 0.06), and large (η^2^_p_ = 0.14) effects [43].

## 3. Results

### 3.1. Acquisition

#### 3.1.1. Shoulder-elbow coordination.

**Fig 4:**
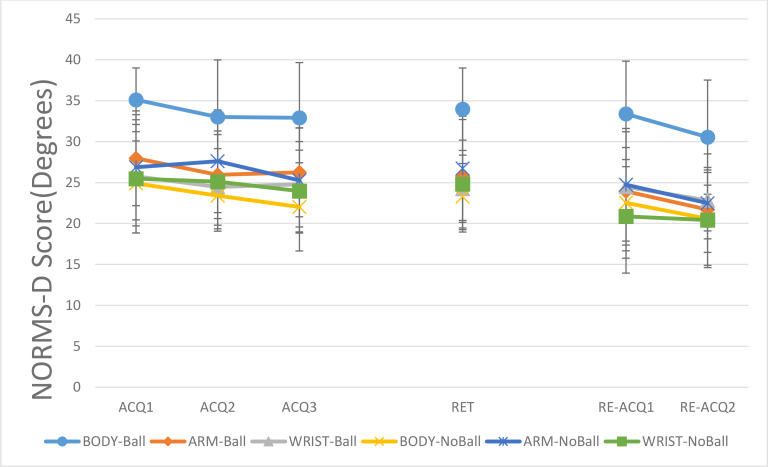
NORMS-D scores for shoulder-elbow coordination across all blocks (ACQ1–3: Acquisition 1–3; RET: Retention; RE-ACQ1–2: Re-acquisition) for all groups (BODY: full-body, 17 markers; ARM: right-arm, 4 markers; WRIST: wrist-only, 1 marker; Ball: throwing to a target; No-Ball: focusing on movement form). Error bars represent the Standard Deviation (SD). Post-hoc tests showed: Groups performed more like the model in ACQ3 vs. ACQ1 and RE-ACQ2 vs. ACQ3/RE-ACQ1; In ball condition, ARM and WRIST outperformed BODY across all phases; BODY-no ball outperformed BODY-ball across all phases.

The NORMS-D data for shoulder-elbow coordination for ball and no ball groups are presented in Fig 4. In acquisition, the main effects of model, F (2, 42) = 1.29, P = 0.28, η^2^_p_ = 0.05 and task constraints, F (1, 42) = 3.63, P = 0.06, η^2^_p_ = 0.08 were not significant. The main effect of block, F (2, 84) = 2.93, P = 0.05, η^2^_p_ = 0.06, was significant. The post- hoc test showed a significant difference between ACQ1 (M = 27.7, SD = 7.3) and ACQ3 (M = 25.9, SD = 7.4), P < 0.05. In general, participants performed more like the model in ACQ3 than ACQ1. The interaction effect of model and task constraints was significant, F (2, 42) = 3.33, P = 0.04, η^2^_p_ = 0.13, other interaction effects were not significant, all Fs < 1. Post- hoc test for interaction showed that in the ball condition, the ARM (M = 26.7, SD = 4.6) and WRIST (M = 25.01, SD = 7.9) groups performed more like the model than the BODY (M = 33.7, SD = 5.1) group, all Ps < 0.05. Also, a significant difference found between the ball (M = 33.7, SD = 5.1) and no ball (M = 23.4, SD = 6.9) conditions in BODY groups, P < 0.05.

#### 3.1.2. Wrist-elbow coordination.

**Fig 5:**
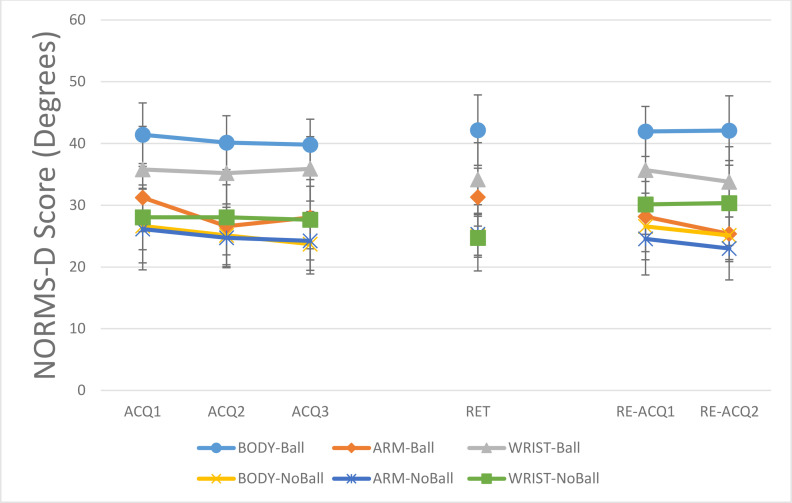
NORMS-D scores for wrist-elbow coordination across all blocks (ACQ1–3: Acquisition 1–3; RET: Retention; RE-ACQ1–2: Re-acquisition) for all groups (BODY: full-body, 17 markers; ARM: right-arm, 4 markers; WRIST: wrist-only, 1 marker; Ball: throwing to a target; No-Ball: focusing on movement form). Error bars represent the Standard Deviation (SD). Post-hoc tests showed: Groups performed closer to the model in ACQ2–ACQ3 vs. ACQ1; In ball condition, ARM outperformed BODY (acquisition, retention) and both BODY and WRIST (re-acquisition); No-ball groups outperformed ball groups (acquisition, retention); BODY-no ball outperformed BODY-ball (re-acquisition).

Fig 5 shows the mean NORMS- D scores for all groups as a function of blocks for the ball (A) and no ball (B) groups. In acquisition, the main effects of model, F (2, 42) = 3.7, P = 0.03, η^2^_p_ = 0.15, task constraints, F (1, 42) = 21.35, P < 0.0001, η^2^_p_ = 0.33, and block, F (2, 84) = 3.13, P = 0.04, η^2^_p_ = 0.06, were significant. The post-hoc test for the main effect of the block showed significant differences between ACQ1 (M = 31.5, SD = 9.08) and AQC2 (M = 30, SD = 9.05), P < 0.05, and ACQ1 (M = 31.5, SD = 9.08) and ACQ3 (M = 29.9, SD = 8.8), P < 0.05. Groups enhanced their similarity to the model in ACQ2 and ACQ3 than ACQ1. The interaction effect of model and task constraints was significant, F (1, 42) = 3.19, P = 0.05, η^2^_p_ = 0.13. Other interaction effects were not significant, all Fs < 1. The post- hoc analysis for the interaction showed that the differences between models (BODY, ARM, and WRIST) were only significant in ball condition. The ARM (M = 28.6, SD = 5.5) group performed significantly, P < 0.05, more like the model than BODY (M = 40.5, SD = 5.5) group. No significant difference was observed between BODY (M = 40.5, SD = 5.5) and WRIST (M = 35.6, SD = 10.1) groups, P > 0.05, and ARM (M = 28.6, SD = 5.5) and WRIST (M = 35.6, SD = 10.1) groups, P > 0.05. Also, participants in no-ball condition (M = 26.03, SD = 5.9) performed more like the model than ball condition (M = 34.9, SD = 8.4), P < 0.05.

#### 3.1.3. Wrist peak velocity.

**Fig 6:**
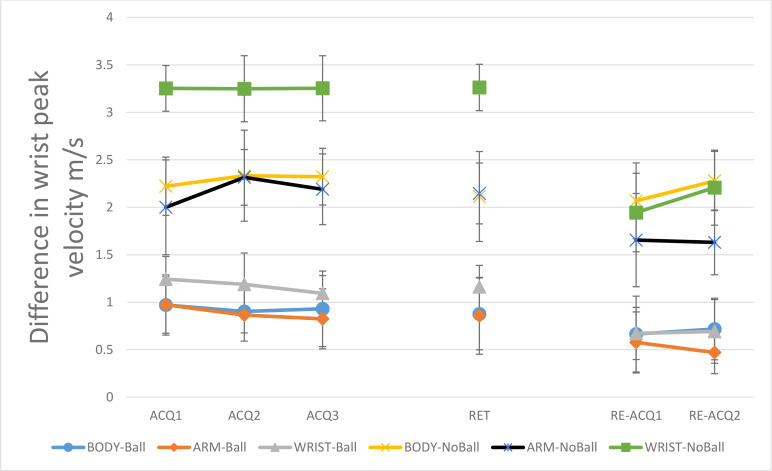
Mean absolute difference in wrist peak velocity (m/s) relative to the PLD model across all test blocks (ACQ1–3: Acquisition 1–3; RET: Retention; RE-ACQ1–2: Re-acquisition) for all groups (BODY: full-body, 17 markers; ARM: right-arm, 4 markers; WRIST: wrist-only, 1 marker; Ball: throwing to a target; No-Ball: focusing on movement form).. Error bars represent the Standard Deviation (SD). The post-hoc tests showed: BODY and ARM groups performed more like the model (acquisition, retention); Ball groups outperformed no ball groups (acquisition, retention); No group difference during the re-acquisition phase.

Fig 6 shows the absolute differences of wrist peak velocity relative to the model as a function of the block for the ball (A) and no ball (B) groups. In acquisition, the main effect of model, F (2, 42) = 6.49, P = 0.003, η^2^_p_ = 0.23, and task constraints, F (1, 42) = 86.21, P < 0.05, η^2^_p_ = 0.67, were significant. Generally results of the post-hoc test for the main effect of the model showed that the WRIST groups (M = 2.21, SD = 1.13) performed less like the model than BODY (M = 1.61, SD = 0.95) and ARM (M = 1.52, SD = 0.92) groups. The ball groups (M = 0.99, SD = 0.43) performed more like the model than no-ball groups (M = 2.57, SD = 0.83). The main effect of the block was not significant, F < 1. The model × task constraints interaction effect was not significant, F (2, 42) = 2.42, P = 0.19, η^2^_p_ = 0.09. Also, other interaction effects were not significant, all Fs < 1.

#### 3.1.4. Outcome error.

**Fig 7:**
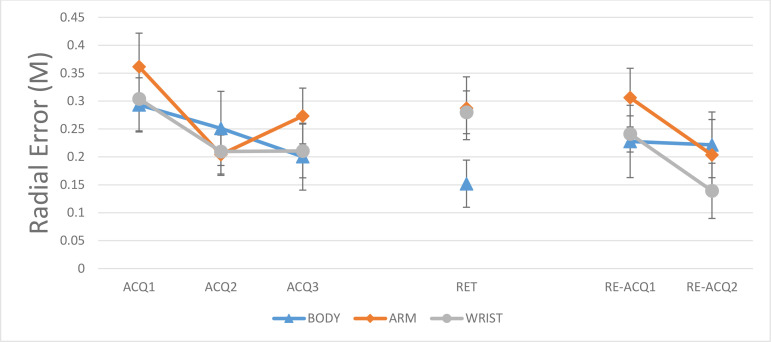
Radial error for demonstration models BODY: full-body, 17 markers; ARM: right-arm, 4 markers; WRIST: wrist-only, 1 marker) across all blocks (ACQ1–3: Acquisition 1–3; RET: Retention; RE-ACQ1–2: Re-acquisition).. Error bars represent the Standard Deviation (SD). Post- hoc tests showed: groups performed more accurate in ACQ2 and ACQ3 than ACQ1; The BODY group performed more accurate than the ARM and WRIST groups (retention); No significant difference between groups in re-acquisition.

In Fig 7, the outcome errors (radial error) are depicted as a function of test periods and demonstration models. In the acquisition, the main effect of the model was not significant, F < 1. However, significant main effect was observed for block, F (2, 42) = 7.24, P = 0.002, η^2^_p_ = 0.25. Results of the post-hoc test for the main effect of block showed that the first block (M = .319, SD = 0.12) is significantly different than second (M = 0.22, SD = 0.09) and third block (M = 0.22, M = 0.12), all P < 0.05. All demonstration groups enhanced their accuracy in the second and third blocks of acquisition in relation to the first block. The model × block interaction effect was not significant, F < 1.

### 3.2. Retention

#### 3.2.1. Shoulder-elbow coordination.

In retention, the main effects of model, F (2, 42) = 1.51, P = 0.23, η^2^_p_ = 0.06, and task constraints, F (1, 42) = 2.52, P = 0.12, η^2^_p_ = 0.05, were not significant. However, the interaction effect of model and task constraints was significant, F (2, 42) = 3.88, P = 0.02, η^2^_p_ = 0.15. The post-hoc test for interaction showed that in ball condition, the ARM (M = 25.83, SD = 7.8) and WRIST (M = 24.25, SD = 8.1) groups performed more like model than the BODY (M = 34, SD = 5.03) group, all Ps < 0.05. Also, a significant difference was found between BODY-ball (M = 34, SD = 5.03) and BODY-no-ball (M = 23.3, SD = 4.04) groups, P < 0.05. Participants in no-ball conditions performed more similar to the model than ball conditions.

#### 3.2.2. Wrist-elbow coordination.

In retention there was a significant difference between demonstration groups, F (2, 42) = 3.36, P = 0.04 η^2^_p_ = 0.13, and the difference between ball and no-ball conditions was also significant, F (1, 42) = 38.21, P < 0.001, η^2^_p_ = 0.47. A Model× Constraints interactions effect was also seen, F (2, 42) = 3.49, P = 0.03, η^2^_p_ = 0.14. This was due to differences in the ball throwing condition only. The ARM (M = 31.3, SD = 6.6) and WRIST (M = 34.17, SD = 8.9) groups performed the throwing action more like the model than the BODY (M = 42.16, SD = 6.7) group. Also, among all demonstration groups (BODY, ARM, and WRIST), the no-ball groups (M = 25.02, SD = 3.9) had a closer approximation to the models’ action than ball groups (M = 35.88, SD = 8.5).

#### 3.2.3. Wrist peak velocity.

In retention, significant main effects were observed for model, F (2, 42) = 6.15, P = 0.005, η^2^_p_ = 0.22, and task constraints, F (1, 42) = 64.27, P < 0.05, η^2^_p_ = 0.60. Results of the post-hoc test for the main effect of model showed that similar to the acquisition, the BODY and ARM groups performed significantly different from the WRSIT groups, all P < 0.05. Means comparison showed that the WRIST (M = 2.21, SD = 1.15) groups performed less like the model than two other observed information (BODY: M = 1.49, SD = 0.91; ARM: M = 1.49, SD = 1.05). In addition, the ball groups (M = 0.96, SD = 0.52) performed more like the model than no-ball groups (M = 2.5, SD = 0.93). The model × task constraints interaction effect was not significant, F (2, 42) = 2.12, P = 0.13, η^2^_p_ = 0.09.

#### 3.2.4. Outcome error.

In retention, the main effect of model was significant, F (2, 23) = 4.21, P = 0.02. The post -hoc test showed significant differences between BODY and ARM, P < 0.05, and BODY and WRIST, P < 0.05, groups. The BODY (M = 0.15, SD = 0.7) group was more accurate than ARM (M = 0.28, SD = 0.12) and WRIST (M = 0.27, SD = 0.10) groups.

### 3.3. Re-acquisition

#### 3.3.1. Shoulder-elbow coordination.

For re-acquisition, the main effect of model was not significant, F (2, 42) = 2.01, P = 0.14, η^2^_p_ = 0.08. The main effects of task constraints, F (1, 42) = 6.004, P = 0.01, η^2^_p_ = 0.12, and block, F (2, 84) = 4.80, P = 0.01, η^2^_p_ = 0.10, were significant. Results of the post-hoc test for the main effect of block showed that the groups performed more like the model in RE-ACQ2 (M = 23.1, SD = 6.7) than ACQ3 (M = 25.9, SD = 7.4) and RE-ACQ1 (M = 25, SD = 8.4), all Ps < 0.05. The interaction effect of model and task constraints was significant, F (2, 42) = 3.58, P = 0.03, η^2^_p_ = 0.14, other interaction effects were not significant, all Fs < 1. The results of Post-hoc test for interaction effect was similar to the retention. In the ball condition, the BODY group (M = 32.29, SD = 6.90) deviated more from the model’s performance than both the ARM (M = 23.96, SD = 6.02) and WRIST (M = 24.04, SD = 6.02) groups, with all differences reaching statistical significance (P < .05). In addition, a significant difference was observed between BODY-ball and BODY-no ball groups, P < 0.05. The BODY-no ball group (M = 21.71, SD = 5.3) performed more similar to the model than BODY-ball (M = 32.29, SD = 6.9) group.

#### 3.3.2. Wrist-elbow coordination.

In reacquisition, the main effect of model, F (2, 42) = 6.32, P = 0.004, η^2^_p_ = 0.23, and the main effect of task constraints, F (1, 42) = 19.16, P < 0.001, η^2^_p_ = 0.31 were significant. The main effect of the block was not significant, F < 1. Significant interaction effect of model × task constraints was observed, F (2, 42) = 4.24, P = 0.02, η^2^_p_ = 0.16. Similar to the retention results, post hoc tests for the interaction effect indicated that the observed differences were attributable solely to the ball-throwing condition. The ARM group (M = 27.18, SD = 6.08) performed more similar to the model than BODY (M = 41.28, SD = 4.04) and WRIST (M = 35.13, SD = 7.9) groups (see Fig 5), all P < 0.05. The BODY–ball group exhibited greater deviation from the model’s performance compared to the BODY–no-ball group (M = 41.28, SD = 4.04 vs. M = 25.12, SD = 5.06), P < 0.05. Other interaction effects were not significant, all Fs < 1.

#### 3.3.3. Wrist peak velocity.

During reacquisition, the main effect of the model was not significant, F (2, 42) = 2.32, P = 0.11, η^2^_p_ = 0.10, but the main effect of task constraints was significant, F (1, 42) = 78.96, P < 0.05, η^2^_p_ = 0.65, and the ball groups continued to perform more like the model than no ball groups. Significant main effect for block was also observed, F (2, 84) = 31.81, P < 0.05, η^2^_p_ = 0.43. The interaction of model × task constraints was not significant, F < 1. However, the interaction effects of model × block, F (4, 84) = 4.79, P = 0.002, η^2^_p_ = 0.18, and task constraints × block, F (2, 84) = 3.99, P = 0.02, η^2^_p_ = 0.08, were significant. Post- hoc test for the interaction effects showed that in the last block of acquisition WRIST groups (M = 2.17, SD = 1.2) performed less like the model than BODY (M = 1.62, SD = 0.98) and ARM groups (M = 1.5, SD = 0.94), all P < 0.05, but there was no significant difference between demonstration groups during reacquisition, all P > 0.05. The interaction effect of model × task constraints × block was not significant, F (4, 84) = 2.23, P = 0.07, η^2^_p_ = 0.09.

#### 3.3.4. Outcome error.

During re-acquisition, the main effects of model, F (2, 21) = 1.24, P = 0.3, η^2^_p_ = 0.10, and the main effect of block, F (2, 42) = 2.31, P = 0.11, η^2^_p_ = 0.09, were not significant. Also, the model × block interaction effect was not significant.

## 4. Discussion

The main aim of this study was to examine the importance of relative motion information under different task constraints. It was hypothesized that participants who were able to observe relative motion information would show a closer intra-limb coordination pattern to the PLD model than groups who did not observe this information. It was expected that relative motion information would have a greater influence under conditions that only required the learner to imitate the PLD model’s movement rather than reaching to an external goal (i.e., throw a ball) [22,23,26]. Also, a reacquisition period allowed testing if relative motion is used later in acquisition.

For shoulder-elbow coordination, in ball condition, the BODY group performed less like the PLD model than the ARM and WRIST groups. Also, the BODY-no ball group performed more similar to the model than BODY-ball group. There was no significant difference between ball and no-ball conditions in other demonstration types (ARM and WRIST). In Wrist-elbow coordination, a similar pattern of difference between demonstration types was observed. In ball condition, the ARM and WRIST groups performed more like the PLD model than BODY group. No significant difference was observed between demonstration types in the no-ball condition. As there was no difference between the ARM and WRIST groups. These results show that the endpoint of action could provide sufficient information for later action reproduction. Similar to other studies [25–27], these findings showed the importance of endpoint information for observational learning. From a theoretical perspective, it is suggested that actions are planned based on their end-trajectories or what has been termed the working point [28]. In line with this theory, our findings showed that observing the endpoint information is enough to learn a complex skill like back-hand throwing action.

For the accuracy measure, it was shown that the BODY group performed more accurate than ARM and WRIST group. At the first look, the results of coordination and the accuracy measures are controversial. But a deeper look at these results provides the possible reason for the weak performance of the BODY group in coordination measure. It is possible that additional relative information within BODY demonstration led to a perception of a simple throwing action, rather than the special backhand throwing action, and by this, focused on reaching the action goal than reproducing the movement pattern. This may have induced an external focus of attention, directing participants toward the action goal rather than the movement pattern consistent with goal-directed imitation theory [38]. Previous research has demonstrated that an external focus can facilitate achievement to the task’s goal [44], with participants often directing their gaze behavior toward external cues [45]. ARM and WRIST groups, with fewer markers, maintained an internal focus on movement patterns, improving coordination. This argument is supported by the higher accuracy of the BODY group than ARM and WRIST groups. Another possible reason can be the higher cognitive load during observation the full-body information. According to the cognitive load theory, the cognitive capacity in working memory is limited, so that if a learning task requires too much capacity, learning will be hampered [46]. Providing a lot of information during full-body demonstration and requirement to achieve an external target can exert too much cognitive load to the learner and distract him/her from the important information to imitate the movement pattern. As a result, the participants in this group focus more on reaching to the external target than imitating the movement pattern. Alternatively, ARM (4 markers) and WRIST (1 marker) conditions, with focused cues, reduce cognitive load, facilitating better shoulder-elbow and wrist-elbow coordination, as supported by studies showing simplified demonstrations improve motor learning [31].

In addition, providing full-body relative information during reacquisition did not result in enhancing similarity of coordination to the PLD model in WRIST and ARM groups. These findings provide partial support for the visual perception perspective view [10] for actions similar to those used in this study, as it is showed that partial relative information is sufficient for action reproduction. However, without eye-tracking data, it is not possible to suggest such a conclusion. Maybe the participants in the ARM group used the endpoint information (wrist) to reproduce the action, as this information was available in the ARM demonstration. The eye-tracking data provide information related to the observed information during acquisition and combining these data with the kinematics data (coordination) can help to understand the role of different types of information in observational learning. The studies using the eye-tracking data showed that observers were more focused on the ARM data during a basketball jump shot [32]. However, eye tracking research cannot provide information related to peripheral vision. Participants may use relative information for action reproduction using peripheral vision. Then a better method to explore the role of information during action observation should consider the role of this type of vision. Also, consistent with the visual perception perspective view [10], providing relative information in the later stages of the acquisition process in this study did not enhance coordination learning. Testing these findings in a more realistic dynamic action in the fields would be interesting.

Besides, these results consistent with the previous studies [33,35,36,47] provided partial support for the goal-directed theory of imitation [38]. The results showed that BODYball group performed less like the PLD model than BODY-no ball group in the shoulder-elbow coordination. Also, in the Wrist-elbow coordination, the no-ball groups performed more like the PLD model than ball groups. Previous studies showed that imitation of skill in no-goal condition results in better reproduction of movement pattern than goal-directed conditions [33,36,47]. However, in this study there was no difference between ARM and WRIST groups in ball and no-ball conditions in shoulder-elbow coordination measure. Maybe the amount of information plays an important role in these conditions, a hypothesis that needs more testing in the future.

In the wrist peak velocity measure, the ball groups showed a closer approximation to the PLD model’s wrist peak velocity than the no-ball groups. The absence of a goal to be achieved has a negative effect on reproducing the control-related parameters [26,48]. In addition, removing the relative motion information had a negative effect on learning the control-related parameters. The WRIST groups had less similarity to the PLD model in the wrist peak velocity measure than two other models (BODY and ARM). This argument was confirmed by the results of reacquisition stage. In this stage, a model × block interaction effect was observed. Results showed that adding full-body information in this stage results in an enhancement of the similarity of control-related parameters of WRIST groups to the PLD model.

The partial eta-squared values from the mixed-design ANOVAs indicate moderate-to-large effect sizes (Cohen, 1988), highlighting the substantial impact of kinematic information and task constraints on observational learning outcomes. In retention, the large effect sizes for task constraints in wrist-elbow coordination (η²_p_ = 0.47) and wrist peak velocity (η²_p_ = 0.60) suggest that the presence of a ball significantly enhances learning of dynamic control parameters, as ball groups outperformed no-ball groups in velocity, likely due to feedback from object interaction. Similarly, the large effect sizes for model-task constraints interactions in shoulder-elbow (η²_p_ = 0.15) and wrist-elbow coordination (η²_p_ = 0.14) indicate that focused kinematic cues (ARM, WRIST) substantially improve coordination learning compared to full-body cues, particularly in the ball condition, suggesting that simplified visual information aids novices in mastering movement patterns. In re-acquisition, the large effect sizes for wrist-elbow coordination (model: η²_p_ = 0.23; task constraints: η²_p_ = 0.31; interaction of model× task constraints: η²_p_ = 0.16) and wrist peak velocity (task constraints: η²_p_ = 0.34) reinforce the importance of arm-specific cues and object interaction, with the ARM group’s superior performance in the ball condition highlighting practical benefits for targeted training. The smaller effect size for the task constraints× block interaction in wrist peak velocity (η²_p_ = 0.08) suggests that differences diminish with practice, indicating that observing relative information after the initial part of practice, may reduce the impact of initial constraints. These effect sizes highlight the importance of aligning visual information and task requirements with specific motor learning objectives, such as using focused cues for coordination and realistic tasks for velocity control in skill acquisition.

## 5. Conclusion

In conclusion, this study showed that restricted relative information or even absolute information is sufficient for learning intra-limb coordination during a goal-directed task. Although, endpoint dynamics can be used to learn intra-limb coordination; this study showed that relative whole-body motion information aids learning the control-related parameters (wrist peak velocity). Also, these results showed that imitation in the absence of an external goal would enhance learning the intra-limb coordination [38] and in some cases (when full-body information is provided), imitation of a goal-directed task can negatively affect the learning of the intra-limb coordination. However, imitation of control-related parameters is more accurate in goal-directed conditions than non-goal directed conditions. Besides, the results of this study showed that providing relative information in later stages of acquisition has no positive effect on the learning of intra-limb coordination, but its positive effect on learning control-related parameters has been observed. Theoretically, these findings suggest that focused relative motion or endpoint information is more effective for coordination learning in complex tasks, challenging the visual perception perspective’s emphasis on comprehensive relative motion. Practically, coaches should use focused demonstrations (ARM, WRIST) for teaching movement coordination in early learning and full-body demonstrations for tasks prioritizing accuracy, optimizing observational learning strategies.

This study has certain limitations that could affect how broadly its findings can be applied. Female participants were not included in this study, and all of the participants were students. This may reduce the generalizability of the results of this study. Given that female participants may possess a distinct motor repertoire compared to their male counterparts—a factor that could influence familiarity with the movement pattern—the inclusion of female participant can affect the finding of the current study. Future research should consider systematically examining this issue by recruiting both male and female participants to explore potential gender-related differences. Also, in no-ball groups, we did not address the action outcome and the movement coordination was the only measure of interest. Although this was to explore the effect of task constraints on acquiring the coordination pattern during action observation, the dissociation between coordination patterns and outcomes might be problematic. In addition, in this study the acquisition phase had a limited number of trials (20 trials) and this may limit the generalizability of these findings. It is recommended that future research use a longer acquisition period (more than one day) and higher number of trials. Also, future research can test the role of different types of information in observational learning using eye-tracking data in male and female participants. In addition, since we employed a novel action and the model was trained on this action for only 1000 trials, it is possible that the model did not learn the action skillfully. This limited training duration could have resulted in a suboptimal point-light display demonstration, potentially influencing the quality of observational learning and the study’s findings. Future research should employ models with more extensive training or established expertise to enhance the validity of the demonstrated movement. Since a pre-test was not included, we were unable to compare the retention data with a baseline (prior to starting the practice) to assess improvements resulting from the practice. Therefore, incorporating a pre-test in future studies would be beneficial. Also, apart from these more theoretical implications of our results, these findings may also have practical consequences. During learning a goal-directed task, coaches or practitioners can provide restricted relative/or absolute information for their learners as it helps higher learning than full-body information. Also, to teach the coordination of a goal-directed action, it is recommended to exclude the need for achievement to the external.

## Supporting information

S1 File Arm file. Point-light display (PLD) showing the movement of the throwing arm during the throwing action, with markers placed on the shoulder, elbow, wrist, and hand. full Body file. Point-light display (PLD) showing full-body movement during the throwing action, captured using 17 markers positioned across key anatomical landmarks. Wrist file. Point-light display (PLD) showing the movement of the throwing arm during the throwing action, with a focus on markers located on the shoulder, elbow, wrist, and hand.
(ZIP)
